# Neurogenic Bowel and Management after Spinal Cord Injury: A Narrative Review

**DOI:** 10.3390/jpm12071141

**Published:** 2022-07-14

**Authors:** Gianna M. Rodriguez, David R. Gater

**Affiliations:** 1 Department of Physical Medicine and Rehabilitation, University of Michigan College of Medicine, Ann Arbor, MI 48108, USA; 2 Department of Physical Medicine and Rehabilitation, University of Miami Miller School of Medicine, Miami, FL 33136, USA; dgater@miami.edu; 3 Christine E. Lynn Rehabilitation Center for the Miami Project to Cure Paralysis, Miami, FL 33136, USA; 4 The Miami Project to Cure Paralysis, University of Miami Miller School of Medicine, Miami, FL 33136, USA

**Keywords:** Spinal cord injury, tetraplegia, paraplegia, neurogenic bowel, bowel incontinence

## Abstract

People with spinal cord injury (SCI) suffer from the sequela of neurogenic bowel and its disabling complications primarily constipation, fecal incontinence, and gastrointestinal (GI) symptoms. Neurogenic bowel is a functional bowel disorder with a spectrum of defecatory disorders as well as colonic and gastrointestinal motility dysfunction. This manuscript will review the anatomy and physiology of gastrointestinal innervation, as well as the pathophysiology associated with SCI. It will provide essential information on the recent guidelines for neurogenic bowel assessment and medical management. This will allow medical providers to partner with their patients to develop an individualized bowel plan utilizing a combination of various pharmacological, mechanical and surgical interventions that prevent complications and ensure successful management and compliance. For people with SCI and neurogenic bowel dysfunction, the fundamental goal is to maintain health and well-being, promote a good quality of life and support active, fulfilled lives in their homes and communities.

## 1. Neurogenic Bowel after Spinal Cord Injury

It is well established that neurogenic bowel dysfunction significantly impacts the quality of life and health of individuals with spinal cord injury (SCI) [1,2,3,4,5,6,7,8]. Neurogenic bowel dysfunction is accompanied by lower gastrointestinal (GI) symptoms such as loss of voluntary control over bowel movements, poor awareness of unintended passage of stool, and difficulty with stool evacuation [4,6,7]. Corresponding upper GI symptoms such as abdominal pain or discomfort, bloating, epigastric burning, and early satiety are frequent [1,7]. Furthermore, GI issues in patients with SCI have been reported to worsen with time and have been shown to contribute to a significant decline in health and wellness, as well as increased hospital admissions [1,2]. 

There are considerable psychological concerns along with the physiologic comorbidities, including depression, anxiety, and fear of bowel incontinence, which can significantly limit an individual’s ability to engage in activities outside their home [2,4,9,10]. As many as 60% of individuals in one study reported adverse effects on life activities, altering their lifestyle due to irregular bowel movements associated with problems of recurrent abdominal discomfort, constipation, and/or fecal incontinence requiring treatment [4]. To facilitate education for all, the PVA Clinical Practice Guidelines for neurogenic bowels have recently been updated by the Consortium for Spinal Cord Medicine [11] and have affirmed/complemented the recent neurogenic bowel guidelines put forward by the Association of the Scientific Medical Societies in Germany [12].

In preparing this manuscript, a review of the literature was performed to identify the most updated physiological and diagnostic information regarding neurogenic bowel after SCI. When available, we utilized the most recent guidelines on the management of neurogenic bowel to describe management and surveillance strategies. We included studies examining interventions and complication outcomes, as well as systematic reviews to provide the most up-to-date information and guidance possible. Additionally, we included studies that focused on quality-of-life metrics to present the patient’s perspective.

## 2. Gastrointestinal Innervation

**Figure 1:**
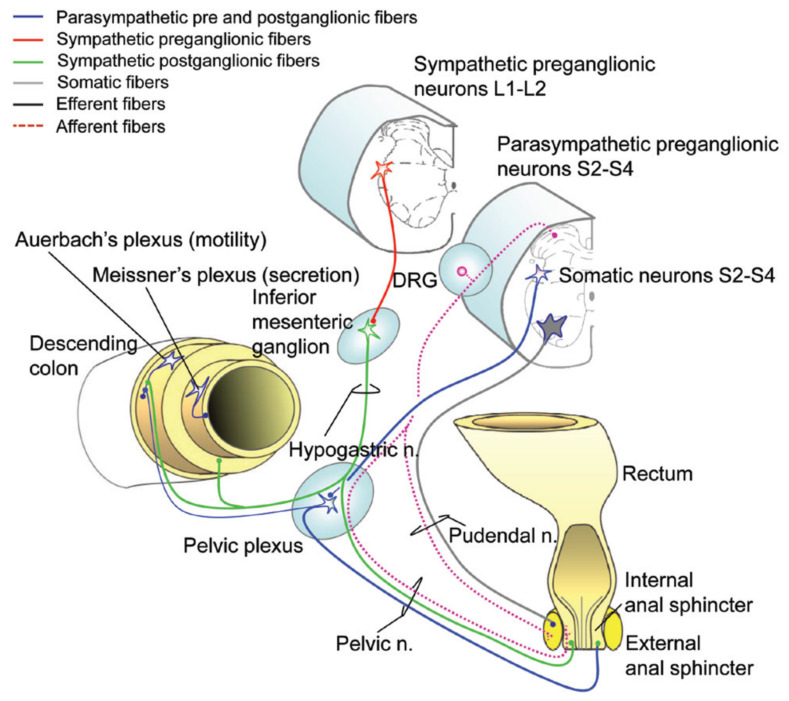
Innervation of the distal gastrointestinal tract. DRG: dorsal root ganglion. Modified by American Spinal Injury Association with permission from Inskip, J.A., et al. Spinal Cord. 2009;47:2-35 [5]. Reprinted with permission from Ref. [14] Topics in Spinal Cord Injury Rehabilitation, International Standards to document Autonomic Function following SCI (ISAFSCI). Copyright 2021 by American Spinal Injury Association.

The GI tract is intrinsically innervated by the enteric nervous system (ENS) which consists of Auerbach’s intramuscular myenteric plexus and Meissner’s submucosal plexus [13]. Activity within the ENS can be modified by portions of the sympathetic nervous system (SNS), parasympathetic nervous system (PNS), and somatic nervous system; normal defecation requires coordination between each entity (Figure 1) [13,14]. SNS innervation to the upper GI tract is provided by the superior and inferior mesenteric ganglia that arise from the T9–T12 preganglionic cell bodies residing in the intermediolateral horns of the spinal cord at those segmental levels. SNS innervation to the descending colon and rectal vault is provided by the hypogastric nerve that arises from the T12–L3 segments of the spinal cord [13,14]. Of note, SNS vascular innervation to most of the small and large bowel is mediated through the greater splanchnic nerve that arises from the T7–T8 spinal cord segments. SNS activation is increased during situations of crisis and GI function is reduced during these periods, shunting blood and substrates to working skeletal muscles to optimize crisis management. PNS innervation to the upper GI tract through the mid-transverse colon is mediated through the vagus nerve (CN-X), whereas the remainder of the large bowel, including the internal anal sphincter, receives PNS innervation from the pelvic nerves that arise from the S2–S4 segments of the spinal cord [13,14]. PNS activation increases during periods of replenishment following the crisis utilization of stored substrates. Somatic innervation (and hence voluntary control) of the external anal sphincter, pelvic floor musculature, and puborectalis muscle occurs via the pudendal nerve that arises from the S2–S4 spinal cord segments. Gut reflexes normally assist with voluntary defecation and include: (1) the gastrocolic reflex mediating colonic contraction in response to stomach stretch receptors; (2) the colocolonic reflex mediating colonic contraction in response to colon stretch receptors; (3) the rectocolic reflex mediating colonic contraction in response to rectal vault stretch receptors; and (4) the anorectal reflex mediating rectal vault contraction in response to anal stretch receptors [11,15]. When the nervous system is intact, these reflexes may be voluntarily suppressed by supraspinal inhibition and continence maintained through the voluntary contraction of the external anal sphincter as well as puborectalis and pelvic floor musculature. Of note, the conus medullaris is the terminal portion of the spinal cord and contains the anterior horn cells of the sacral segments, S2–S5. Injury to this region of the spinal cord often involves both upper and lower motor neurons, as the exiting peripheral sacral nerves are likely to be damaged. Hereinafter, this will be referred to as conal or subconal SCI, whereas all spinal cord segments above this region will be referred to as supraconal SCI. 

## 3. Pathophysiology of the Neurogenic GI Tract in SCI

SCI results in neurologic dysfunction characterized by the dysmotility of various segments of the gastrointestinal tract (primarily colonic), weakness of the pelvic floor and rectal sphincters, and impaired sensation in the anal and perineal areas [15,16]. Suprasacral SCI typically results in colonic hyperreflexia, which is opposed by the hyperreflexia of the external sphincter, puborectalis and pelvic floor musculature, resulting in rectosphincter dyssynergia and high colonic pressures and constipation with intermittent fecal incontinence [15,16]. Conversely, conal and subconal SCI cause hyporeflexia or flaccidity of colon, rectum, and sphincters, resulting in low pressure but uncontrolled fecal incontinence [17,18]. While both types of SCI can lead to fecal incontinence, hyperreflexic bowels can also cause autonomic dysreflexia (AD), a hypertensive crisis in persons with SCI above T6 due to the uninhibited sympathetic reflex activity mediated along the greater splanchnic nerve in response to noxious stimuli below the level of SCI. Bowel-related stimuli are the second-leading cause of AD (only urological stimuli are more frequent), and may include gastric ulcers, duodenal ulcers, cholecystitis, cholelithiasis, appendicitis, bowel distension, bowel impaction/obstruction, GI instrumentation, bowel care reflexes, and hemorrhoids [19]. AD can be life-threatening and warrants immediate intervention as outlined by other authors [14,19]. Of note, AD from any source can profoundly increase SNS outflow and reduce gut motility. Neurogenic bowel (NB) from SCI is classified under the Rome IV criteria for functional constipation (FC) and defecatory disorders (DDs) [20,21]. Furthermore, other SCI factors exacerbate problems associated with FC and DDs such as decreased mobility, poor nutrition, poor hydration, and use of medications that are known to affect GI motility such as opiates, anticholinergics, and antispasmodic agents [20,21].

### 3.1. Supraconal (Suprasacral) Neurogenic Bowel

As above, hyperreflexic NB patterns of dysfunction occur in SCI above the conal segments of the spinal cord (supraconal). Krogh et al. showed that colonic motility and stool propulsion are affected in SCI using swallowed markers to measure colon transit on serial radiographs [16,22]. Motility was shown to be prolonged for participants with chronic supraconal SCI in the ascending, transverse, descending, and rectosigmoid colon, while total GI transit time averaged 3.93 days (versus 1.76 days for non-SCI controls). Mean total GI transit times were compared for patients with lesions above T9: 2.92 (±2.41) and from T10 down to L2: 2.84 (±1.93) to assess the effects of sympathetic innervation. No significant differences were shown when segmental times were compared. The GI transit time in participants with complete SCI in the acute (5–21 days) and chronic (6–14 months) stages demonstrated greater prolongation in the acute rather than chronic phase. Slower transit throughout the colon was observed but appeared less severe in the rectosigmoid segment [16,22]. Studies on colonic compliance in supraconal SCI have shown either a decrease in colonic compliance (rapid pressure rise and hyperreflexia) or normal colonic compliance [15,17,18,23]. The internal sphincter normally relaxes when rectal distention occurs. The external sphincter normally provides voluntary control over the evacuation of stool but may relax spontaneously when there is significant rectal distention. People with supraconal SCI and hyperreflexic NB typically have normal or increased anal sphincter tone, intact anocutaneous (anal wink), and bulbocavernosus reflexes [15,17,18,23]. The colorectal reflex which remains intact in hyperreflexic NB also activates the hyperreflexic external sphincter, contributing to rectal sphincter dyssynergia and difficulty with defecation [15,17,18,23]. Additionally, the impaired sensory perception of colorectal distension reduces the urge to defecate, amplifying the problem in hyperreflexic NB. For those with incomplete SCI, 43% have some perception of colorectal distention experienced as abdominal discomfort relieved by bowel evacuation [15,17,18,23]. 

### 3.2. Conal/Infraconal Neurogenic Bowel

A hyporeflexic NB pattern of dysfunction that occurs in SCI at or below the conal segments (conal/infraconal) may include injury to the cauda equina, sacral nerves, or the pudendal nerves. There are diminished or absent anocutaneous, bulbocavernosus, and other lumbosacral reflexes [15,16,17,22,23]. The external sphincter innervated by the somatic nervous system and the internal sphincter innervated by the PNS are equally affected with poor reflex activity. Decreased tone and weakness in the pelvic floor muscles and external anal sphincter create the impression of a shortened anal canal and a nonpalpable puborectalis muscle ridge. Fecal incontinence is common due to the flaccid paralysis of these structures. Hyporeflexic NB is exceptionally difficult to manage due to the lack of both PNS and somatic reflex activity [15,16,17,22,23]. A study done using radio opaque marker tests revealed that people with acute and chronic conal/infraconal SCI had prolonged total GI transit times (4.91 days and 3.61 days, respectively) [22]. Consequently, another study completed with scintigraphy showed delayed transit in the descending colon in 38% and in the rectosigmoid colon in 27% of people with conal/infraconal cord lesions; total colon transit times were likewise significantly delayed in these subjects [24]. Poor movement of stool from colonic inertia leads to fecal loading and hard stool which further strain weak and hypotonic sphincters and pelvic floor muscles. Paradoxical liquid incontinence around stool impaction may often occur and occasionally contribute to rectal prolapse [15,16,17,22,23]. 

### 3.3. Constipation and Fecal Incontinence

Constipation and fecal incontinence frequently occur and can present, independently or collectively after SCI, as a consequence of dysfunctional colonic motility and sphincter tone [22,25,26]. Both hyperreflexic and hyporeflexic NB increases the gut transit time which contributes to FC [24,25,26]. Regardless of the pattern of delay, constipation is a result of suspended stool movement throughout the colon, causing hard stools from the reabsorption of water and electrolytes across the intestinal lumen by the inhibition of secretomotor neurons in the ENS; the pattern worsens under SNS activation associated with AD [27]. Likewise, DDs have been equally demonstrated in both hyperreflexic and hyporeflexic NB exemplified by the poor emptying of the rectosigmoid. Rectosphincter dyssynergia causes obstructed defecation with intermittent fecal incontinence in hyperreflexic NB. Conversely, diminished rectal resting pressures and the flaccid paralysis of the anal sphincter and pelvic floor muscles found in hyporeflexic NB fail to facilitate rectal emptying [2,23,28]. In hyporeflexic NB, fecal incontinence can be due to both FC and DDs as a result of poor rectoanal and perineal sensation, lack of voluntary external anal sphincter contraction, and fecal loading with overflow incontinence [2,23,28,29,30].

Abdominal pain and discomfort are not uncommon in NB due to distention and forceful contractions in the GI tract precipitated by chemical/mechanical irritation, ischemia, injury, inflammation, or obstruction [27,31]. Bloating, early satiety, and nausea present frequently in NB in the absence of mechanical obstruction caused by dysmotility and neurologic dysfunction in the GI tract. Conversely, the hypertonicity of the GI tract contributes to these symptoms in the supraconal SCI disinhibition of autonomic, myenteric, or smooth muscle systems, contributing to uncoordinated circular muscle contractions that prevent distal propulsion and cause functional obstruction [27,32]. Problems with dysphagia, gastroparesis, or chronic intestinal/colonic pseudo-obstruction may arise along with anorexia, abdominal pain, diarrhea, and constipation [27,33,34,35].

Diarrhea in the presence of NB is usually related to overflow constipation, but can also be caused by antibiotic use, GI infections, over-activation of secretomotor neurons by histamine from inflammatory and immune mediated cells in the mucosa and submucosa, and/or vasoactive intestinal peptide and serotonin from mucosal enterochromaffin cells. These chemicals in turn affect presynaptic inhibitory receptors, impeding the release of norepinephrine from postganglionic sympathetic fibers that inhibit secretomotor neurons [27,36]. As mentioned above, for persons with SCI above T6, life-threatening AD may result from any of the noxious stimuli listed, although the individual may have no sensation of abdominal pain or discomfort; rapid assessment and intervention is warranted [19].

## 4. Comprehensive Evaluation for Neurogenic Bowel

### 4.1. Medical and GI History 

People with neurogenic bowel will need thorough evaluation beginning with a comprehensive medical history. This should include past medical history, allergies, medications, family history, social environment/resources, and a thorough review of systems, including neurologic diagnoses and functional impairments that might contribute to GI symptoms, bowel, and defecation problems [11,12,37]. It is important to determine the duration, severity, and progression of any GI problems, as well as the current bowel care program for emptying [11,37]. The review should include oral and rectal bowel medications, defecation frequency, stool consistency (using the Bristol Stool Scale) [38], quantity, time of day, and strategies for defecation utilized in the past. It is vital to be aware of the dosage, frequency, and duration of use for medications that might decrease GI motility (e.g., opiates, anticholinergics, antispasmodics, and tricyclic antidepressants) and compound problems related to NB [11,37]. Queries should include the total intake of fluids, diet, physical activity, and any limitations or obstacles to maintaining fecal continence [11,37]. All GI symptoms must be elicited, including AD, abdominal pain or discomfort, abdominal bloating and distention, rectal urgency, impaired sense of urgency, incomplete stool emptying, and episodes of incontinence with or without “stress” (i.e., sneezing, coughing, or transfers) [11,37]. 

The International Standards for Neurological Classification of Spinal Cord Injury (ISNCSCI) are the most validated and reliable measures for determining neurological impairment as medical history is obtained [11,39]. Similarly, the International Standards to document Autonomic Function following SCI (ISAFSCI) should be documented, particularly for individuals with SCI at or above T6 who are at high risk of AD [14]. There are various measures that facilitate the assessment of NB after SCI. For the evaluation of fecal incontinence, the Fecal Incontinence Severity Scale [40], Wexner Continence Scale [41], or St. Mark’s Incontinence Score [42] can be used. For the evaluation of constipation, the Patient Assessment of Constipation-Symptoms (PAC-SYM) [43], Cleveland Constipation Score [44], or Wexner Constipation Score [44] can be used. The International SCI Bowel Function Data Set (ISCIBFDS) and the Neurogenic Bowel Dysfunction Score (NBDS) were specifically created and validated for people with SCI [45]. It must be recognized that quality of life (QOL) is significantly affected by both fecal incontinence and constipation, which FI-QOL [46] and PAC-QOL [47] measure, respectively. Finally, the Spinal Cord Injury-Quality of Life (SCI-QOL) measurement system has the Bowel Management Subscale that can be used for the assessment of the impact of NB dysfunction on the daily living and QOL of people with SCI [48].

### 4.2. Physical Examination

The physical examination is integral in the assessment and management of NB [11,12]. Basic assessment for constitutional signs of malnutrition and dehydration, including loss of weight, pale skin, dry mucous membranes, poor skin turgor, orthostatic hypotension, and tachycardia is necessary. If the person with SCI has never been evaluated for level and completeness of injury, the ISNCSCI and the ISAFSCI should be performed to determine whether NB is hyperreflexic or hyporeflexic [11,14,37,39]. The abdomen should be examined for fullness, wall abnormalities, and asymmetry. Auscultation is performed for altered bowel sounds, rubs, or vascular bruits. Percussion precedes palpation and is helpful for determining underlying gas or fluid distention. Palpation determines the presence of tenderness, ascites, organomegaly, masses, or hard stool. A rectal/pelvic examination is initiated by inspection for hemorrhoids, fissures, or an enlarged anus. Perineal descent with straining and voluntary contraction of the anal sphincter should be observed. The presence of the anal wink and the bulbocavernosus reflexes is pertinent, as hyperreflexia, hyporeflexia, or normal reflexes will help determine appropriate NB management. The digital rectal examination identifies sensory and motor function, in addition to identifying structural abnormalities such as hemorrhoids, rectoceles, or rectal prolapse. The sensory perception of deep anal pressure and/or voluntary anal contraction is indicative of the preservation of neurologic function. Pelvic floor relaxation and the expulsion of the finger with simulated defecation upon bearing down provides significant information on muscle weakness, coordination, and tone. Dyssynergia is present when there is paradoxical contraction of the sphincter and pelvic floor with rectal contraction, and typically occurs in hypertonic muscles. Incomplete anal contraction is associated with sphincter and pelvic floor weakness [37,49,50]. 

### 4.3. Laboratory

The most fundamental and relevant information for the neurogenic bowel is derived from history and physical examinations [11,12]. Further testing is warranted when GI problems are acute, progressive, when causality is unclear, when history is not reliable, or when conservative management has been unsuccessful and surgical options are being entertained. Blood tests are necessary when anemia, infection, dehydration, or malnutrition are suspected. Stool sampling is performed to evaluate for cancer, infection, or parasites [11,37].

### 4.4. Imaging

The simplest radiologic test is an abdominal X-ray for the evaluation of fecal loading, impaction, megacolon, intestinal obstruction, or perforation [49,51,52]. If more information is necessary, an abdominal CT scan can delineate gastric, small intestinal, colonic, or pelvic structural or anatomical abnormalities [11,12]. CT is mainly utilized to identify small or large intestinal obstruction, and can establish the cause, site, and extent of an obstruction. The CT scan assists with determining emergent (i.e., strangulated, or ischemic obstructions) versus non-emergent obstructions (e.g., adynamic ileus) [11,20,21,53,54]. Defecography can be performed when there is high clinical suspicion of structural causes of rectal outlet dysfunction related to rectal prolapse, rectocele, or enterocoele [11,37,55]. Defecography has the advantage of evaluating the anorectum and pelvic floor muscles before, during, and after defecation in real time with the use of fluoroscopy or magnetic resonance imaging (MRI). The response and coordination of the rectum, sphincters, and pelvic floor to the attempted defecation of barium paste instilled in the rectum (to mimic stool) can be closely assessed dynamically [50,52,56]. MRI defecography provides better imaging of the anal sphincter and pelvic floor muscles, specifically the levator ani muscle, and improved resolution of soft tissue structures in the pelvis surrounding the rectum and anal canal, including the bladder, uterus, and small intestine [11,20,21,49,53,54,57]. 

### 4.5. GI Transit Time

Defecation problems can contribute to and result in prolonged colonic motility. Colonic transit time can be evaluated with radiopaque markers, scintigraphy, or a wireless motility capsule [11,12]. It is performed by either swallowing radiopaque markers or taking dye followed by abdominal radiography on multiple days as these markers pass through the segments of the colon (ascending, transverse, sigmoid, and rectum) [22,24,25]. The Wireless Motility Capsule has the benefit of measuring motility in each of the segments of the GI system (gastric, small intestinal, and colonic) and throughout the whole gut [21,58]. The clinical practice guidelines (CPGs) of the American Gastroenterological Association (AGA) and American College of Gastroenterology (ACG) recommend evaluating for the prolongation of colonic transit time for progressive GI complaints and worsening constipation that has been unresponsive to conservative treatment with medications [50,52]. 

### 4.6. Manometry

Impaired defecation in neurogenic bowel resulting in constipation and/or fecal incontinence due to impaired motor and sensory function can be assessed with anal rectal manometry (ARM) [11,12]. A compilation of events may contribute to defecatory dysfunction identified by the ARM [11,20,21,53,54]. Dyssynergia occurs with the paradoxical contraction of the rectal sphincter and pelvic floor muscles during simulated defecation, causing increased pressures in the anal canal with an insufficient increase in rectal and intraabdominal pressures, as well as inadequate propulsive forces. This pattern typically occurs with hyperreflexic NBD [23,28,59]. In contrast, low rectal resting and squeeze pressures occur in hyporeflexic NBD [18,60]. The Balloon Expulsion Test (BET) is usually assessed with the ARM and evaluates pelvic floor and rectal sphincter function by determining the ability and duration of time a balloon-tipped catheter being expelled from the rectum with simulated defecation [16,61]. An electromyographic (EMG) study is an additional way to assess pelvic muscle activity and response through electrodes positioned in bilateral areas of the rectum [50,52,56]. Pudendal nerve conduction studies (NCSs) are usually performed with the EMG study, and can diagnose peripheral pudendal nerve injury [16,23].

People with neurogenic bowel may experience significant issues with upper gastrointestinal symptoms—abdominal pain, discomfort, bloating/fullness, and early satiety—which are typically associated with constipation. However, further investigation for other causes is warranted if constipation has been relieved, a good bowel program is in place, and more serious intestinal obstruction has been ruled out. The Gastric Emptying Study can evaluate for gastroparesis. It measures the rate at which solids and liquids are emptied from the stomach and can identify delay in emptying, which can be the main cause of these symptoms [11,20,21,53,54]. Hydrogen breath testing with either glucose or lactulose can be performed to identify small intestinal bacterial overgrowth syndrome (SIBO) as a cause of these symptoms as well [62]. People with GI motility issues are at high risk for SIBO that, if present, can be successfully treated with antibiotics. Considerable increases in bacteria or methanogens develop in the stomach and small intestine where there typically are low numbers of bacteria [63,64]. For people with a neurologic disease, this is most likely due to upper gastrointestinal dysmotility and an impaired ability to clear undesired bacteria and undigested material [63].

## 5. Management of the Neurogenic Bowel

Once the history and physical examinations have been completed, the clinician should be able to make a diagnosis of supraconal (hyperreflexic) or conal/infraconal (hyporeflexic) NB [11,12,37]. Rehabilitative management puts emphasis on establishing a bowel program (defined as a total management plan for bowel function), and for bowel care (referring to assisted defecation) [11,12]. This personalized approach is based on all the information gathered in the history, physical examination, and diagnostics that includes the utilization of oral and rectal bowel medications, techniques, and devices for rectal emptying, education, supplies and equipment, scheduling, and caregiver requirements [37]. The goals of the NB bowel care program should be clearly defined for the person with SCI and their caregivers to ensure compliance and success. The medical provider, in partnership with the person with SCI and their caregiver(s), should be aware of individual responses to medications and techniques in the setting of diverse habits, lifestyles, and access to resources so that designing the program jointly will be beneficial. Expectations and education should be provided to everyone involved with the understanding that there is no quick fix, and that compliance, consistency, and regularity are most important in achieving the desired goals [37], which include: (1) regular bowel movements (BMs) daily or every other day (at least three times per week); (2) adequate stool outputs per BM (i.e., moderate amount~1.5–2 cups for daily BMs; large amount∼3–4 cups for every other day BMs); (3) complete bowel evacuation at a regular time of day; (4) no episodes of incontinence while limiting stool occurrences to once a day; (5) maintaining soft, formed stool consistency (Bristol Stool Type 4–5) [38] while preventing hard stools (Bristol Stool Type 1–3) [38]; (6) completing bowel care within 30 (ideal)–60 min; and (7) physical or instructional independence with the bowel program/care [37]. 

The initial bowel care program should be implemented immediately upon admission to acute rehabilitation, if not before. The consistent and regular evacuation of adequate amounts of stool daily encourages habituation and prevents severe constipation and fecal impaction, even in the early stages of “spinal shock.” A bowel care program that is timed, planned, and complete promotes control over bowel evacuation, allows predictability, and reduces episodic fecal incontinence [37]. A combination of oral bowel osmotic and stimulant medications in conjunction with rectal medications, techniques (digital stimulation/evacuation), and devices (flushing enemas/transanal irrigation) facilitate regular and complete defecation. Fostering physical and/or instructional independence enables the person with SCI to take ownership of their own NB program/care [37]. To prevent problems that arise from NB and maintain GI health, the basic necessities of the human body must be fulfilled, including nutritious food, adequate fluids, mobility, activity, wellness, as well as reducing or discontinuing constipating medications and supplements [11,12]. 

### 5.1. Medical Management of Functional Constipation in SCI

Being cognizant of the goals for the bowel care program, the most suitable regimen will require the trial and error of miscellaneous medications, dose, duration, frequency, and efficacy. Education and information must be provided about the ever-changing NB and can be affected by various factors, including diet, hydration, activity, illness, aging, and the use of other medications. For this reason, regular medical follow-ups and care will be vital. A high incidence of late GI problems are reported in an initially successfully-managed SCI population [65]. Ultimately, the individual should become independent with these adjustments to meet the jointly established goals of the NB bowel care program [37].

Oral bowel medications are mainly utilized to facilitate the movement of stool throughout the colon and into the rectum to optimize and complete stool evacuation for both the hyperreflexic and hyporeflexic NB [11,12,37]. There are two main categories of oral bowel medications that can be used independently or together. These are the bowel stimulants such as senna (Senokot^TM^), bisacodyl (Dulcolax^TM^), and osmotic agents such as polyethelene glycol (Miralax^TM^), lactulose (Cephulac™), magnesium derivatives (e.g., Milk of Magnesia^TM^, magnesium citrate), and/or stool softeners such as docusate (e.g., Colace^TM^) [20,21,53].

Food and fluid choices affect the consistency of stools and influence the delayed transit times in NB [11,12,37,66]. It is ideal to maintain soft, formed, bulky stools to facilitate movement throughout the colon. Prolonged motility promotes hard stools as a result of increased fluid resorption with ensuing constipation, which implies difficulty moving through the haustra of the colon due to the lack of elasticity, creating a vicious cycle [37,66]. High pressures in the colon from solid stool cause hemorrhoids and diverticula formation in those with SCI [67]. Hard stools also exacerbate persistent straining and can cause pudendal neuropathy at the anal sphincter [67]. High-fiber foods maintain more fluid in the stools, improve bulk and elasticity, and decrease colonic pressures [20,21,66]. The recommendations for total dietary fiber consumption from food is 25–30 g [68]. Fiber must be taken with caution since, when it is consumed incorrectly, it can result in worsening constipation. It is imperative that fluid intake is adequate, i.e., 2.5–3.0 L (water, non-caffeinated liquids) [69] on a high fiber diet to prevent constipation. Conversely, diuresis can occur with highly caffeinated drinks such as coffee, tea, or energy drinks and can result in dehydration [37]. Vegetables, fruits, and grains which provide natural fiber are preferred over supplemental fiber such as psyllium (e.g., Metamucil™, Fiberall™), calcium polycarbophil (e.g., Fibercon™), and methylcellulose (e.g., Citrucel™). There are studies demonstrating constipation resolution with high prune intake (6 prunes 2x daily) attributed to fiber and fructose [57,70]. Hemp seed extracts can also be helpful in some patients [20]. Like other components of NB management, the need and titration for fiber should be personalized and evaluated for each individual [66,68]. The intake of probiotics (e.g., Bifidobacterium lactis DN173010, Lactobacillus casei Shirota, Lactobacillus casei YIT) was found to improve constipation in a recent systematic review; however, these studies are subject to a high risk of bias and results must be used with caution [20,71]. 

Dietary considerations in people with SCI should include the awareness of specific foods that increase gas production. Foods with high fermentable oligosaccharides, disaccharides, monosaccharides, and polyols (FODMAPs) may lead to increased GI symptoms in NB dysfunction [11,72,73]. These foods raise the concentration of fructose in the excess of glucose (apples, pears), lactose (dairy products), fructans (wheat, onions), polyols (artificial sweeteners and sorbitol), and galacto-oligosaccharides (legumes, cabbage) [73]. FODMAPs are short-chain carbohydrates which are poorly absorbed in the small intestine which in turn increases osmotic effects and water in the GI lumen. FODMAPs undergo fermentation by colonic bacteria to short-chain fatty acids and release hydrogen, methane, and carbon dioxide gases which can result in bloating, cramping, abdominal distension, pain, and/or altered bowel movements [11,72,73]. Although studies have not been completed in people with SCI, the low FODMAPs diet has been demonstrated to improve symptoms in individuals with irritable bowel syndrome [72,73,74]. It is most appropriate that an experienced dietician manages, guides, and assists these individuals through the various phases of the low FODMAPs diet [11,72,73]. 

### 5.2. Novel Medications for Constipation

Newer medications to maintain or improve the NB care program can be used when the basic medications listed above have not been effective [20,21,37,53]. Lubiprostone enhances intestinal and colonic transit by increasing intestinal fluid secretion through the activation of type 2 chloride channels, facilitating stool passage. It acts on prostaglandin E receptors which aid gastric and colonic muscle contraction and motility [20,21,53,75,76]. Linaclotide is an agonist of guanylate cyclase-C (GC-C) receptors located on the luminal surface of the intestinal epithelial cells. It improves cGMP conversion to cyclic guanosine monophosphate (cGMP) which enhances a signal transduction cascade, activating the cystic fibrosis transmembrane conductance regulator which results in the secretion of fluid into the lumen and promotes intestinal transit. Plecanatide is a similar drug to Linaclotide with analogous effects [20,21,53,75,76]. Prucalopride is a selective 5-hydroxytryptamine receptor agonist which stimulates colonic transit and improves constipation by causing high amplitude propagated contractions, hence enhancing segmental contractions [20,21,53,77]. Methylnaltrexone and Alvimopan are peripherally acting μ-opioid receptor antagonists which selectively block μ-receptors outside of the CNS and improve constipation related to the use of high dose opioids; they do not reverse analgesia and/or induce opioid withdrawal [20,21,53,78].

### 5.3. Management of Defecation Dysfunction in Hyperreflexic NBD

An advantage of hyperreflexic NBD is that defecation can be initiated by stimulating the defecatory reflex activity with digital stimulation, rectal stimulant medication, enemas, or electrical stimulation [37]. Reflex relaxation of the IAS and the EAS occurs with the use of these listed methods alone or in combination, activating anorectal colonic reflexes, enhancing left colon motility, and facilitating stool evacuation [67,79]. Rectal medications are used to initiate and maintain reflex defecation. The medication is introduced into the rectum 30 min prior to the intended NB program/care, followed by digital rectal stimulation [37]. The available suppositories are vegetable-oil-based bisacodyl (i.e., Dulcolax™), and polyethylene glycol bisect bisacodyl (i.e., Magic Bullet™) and glycerine. Other options include docusate mini-enema (i.e., Enemeez™), small volume enemas such as phosphor-soda enema (i.e., Fleets™), and bisacodyl enema [20,21]. Digital rectal stimulation is completed by introducing a gloved, lubricated finger into the rectum and performing gentle, circular strokes in 20 s intervals every 5–10 min until the rectum is fully cleared of stool [37]. 

### 5.4. Management of Defecation Dysfunction in Hyporeflexic NBD

Pelvic floor and sphincter flaccidity as well as decreased or absent reflexes characterizes the hyporeflexic NBD. The evacuation of stool in hyporeflexic NBD is most effective with disimpaction or flushing enemas (preferably with warm tap water in the range of 500–1000mL) performed once or twice daily [11,12,37]. Ideally, stool consistency should be soft, formed, and bulky due to the high risk of fecal incontinence. The use of oral bowel stimulants and/or osmotic medications to facilitate the movement of stools to the rectum should be used with caution since watery stools can increase episodes of fecal incontinence [20,21,37,53].

### 5.5. Mechanical Interventions

Transanal irrigation is an excellent option for rectal evacuation in both hypperreflexic and hyporeflexic NB. The transanal irrigation device (e.g., Peristeen™, Navina™) includes a rectal balloon catheter and a pump which can provide pulsed irrigation to cleanse the rectum up to the sigmoid [20,21,37]. Multiple studies have shown that it is a safe and effective method to manage hard stools, fecal loading, and fecal impactions [67,80]. Transanal irrigation was demonstrated to improve constipation, incontinence, overall bowel function, total time for bowel care, gastrointestinal symptoms, and quality of life in individuals with SCI compared to the regular bowel program in a large multicenter trial [29,60,80,81]. Subsequent studies revealed lower costs of care [29,60], reduced or discontinued use of medications [60,82], long-term successful outcomes with the continued use of the device, and the resolution of symptoms [67,80]. 

Pelvic Floor Therapy using sensory, mechanical, visual techniques as well as strategies of anorectal and pelvic floor muscle activity may be useful to treat NB in people with incomplete SCI who have partially intact sensory and motor function. Therapy utilizes biofeedback with the goal of retraining muscles to coordinate defecation by increasing intra-abdominal pressure and relaxing the pelvic floor muscles/anal sphincter to improve stool evacuation and emptying. Biofeedback can be performed using electrodes (electromyographic or manometry) applied to the perineum and external anal sphincter in conjunction with balloon expulsion training, sensory relearning with retraining for sensations of rectal filling and movement of pelvic floor muscles, and Kegel exercises [20,21,37,83].

### 5.6. Surgical Interventions

Colostomies are medically indicated in SCI when conservative management has failed or when recurrent bowel impactions/obstructions, severe colonic inertia, or poorly healing pressure injuries due to fecal soiling occur [11,12]. Colostomies have been found to be advantageous in various systematic reviews and many studies on persons with SCI [29,67,84,85,86,87]. These have shown that with a colostomy, bowel emptying is more regular and consistent; bowel care is streamlined and reduces time spent on stool evacuation [67,84,86,87,88]; abdominal pain, discomfort, bloating, and other symptoms improve [67,86]; episodes of incontinence are prevented; serious GI complications are diminished; rates of hospital admissions are reduced [67,86]; and independence is promoted which improves quality of life and allows more activities outside the home and travel [67,86,87,88]. The majority of people with SCI who have had colostomies indicate they should have had the colostomy performed earlier and do not regret having had the surgery. A colostomy should be recommended sooner in people who have had significant difficulties with maintaining bowel health with conservative management [67,84,88]. A left-sided sigmoid colostomy is usually proposed rather than a more proximal diversion or an ileostomy, since this is more likely to produce formed stools and prevent dehydration [67,89]. 

The Malone Antegrade Continence Enema (ACE) procedure can be approached when bowel care is severely protracted, when conservative management has failed, or when recurrent bowel impactions/obstructions have occurred. The ACE system involves surgery that creates a catheterizable appendicocecostomy stoma, where the appendix lumen is brought to the right lower abdominal wall and a stoma is created for access with a catheter to use for routine antegrade enema cleansing of the colon [90]. Currently, this procedure can be performed laparoscopically [91]. For people who have exceptionally slow colonic transit, an alternate surgical procedure, the Macedo–Malone or left-sided ACE (LACE) can be performed where a portion of the descending colon is used to create a catheterizable tube attached to the left lower abdominal wall [92]. The ACE flushes the whole colon, since this is positioned in the proximal colon, and can be performed 2–3 times a week. The LACE only flushes the more distal descending colon with less cleansing, but should be performed more often, i.e., daily or every other day. The advantage of the LACE is that the catheterizable stoma can be filled with 200–600 mL of tap water to induce propulsive colonic peristalsis and defecation within 10–20 min, as opposed to the ACE in which larger amounts of water are required for flushing and can take 1–2 h to complete [92]. 

## 6. Conclusions

In recent years, the importance of translational and clinical bowel research for SCI has been significantly advocated, with clear recommendations for the development and utilization of clinical assessment tools and interventional strategies offered to improve the care and quality of life for those living with neurogenic bowel [55,93]. Of note, this narrative review incorporates the most recent SCI-specific bowel care guidelines [11,12], as well as updates on autonomic dysfunction after SCI [14,19] that have not been included in other recent reviews [94]. As medical providers, we want to ensure that our patients with SCI remain healthy, well and away from the hospital, and without illness as best as possible. We will need to form close partnerships with our patients and educate them to achieve this. We are all aware that dealing with NB is very challenging, and entails setting expectations, patience, close monitoring, and follow-up. We need to consider each person’s unique circumstances for successful medical management and enhanced compliance. Our primary goal is for our patients to have the best quality of life they can have. We all have to work with and optimize what we have in our toolbox as of right now for our approach of NB evaluation and management. Further research and study of NB is necessary now and in the future, to be able to provide more responsive ways to address the demands for better evaluation, medical management, and provision of care. 

## Data Availability

Not applicable.
